# Bioactivity guided isolation of cytotoxic terpenoids and steroids from *Premna serratifolia*

**DOI:** 10.1080/13880209.2017.1301491

**Published:** 2017-03-19

**Authors:** Mahesh Biradi, Kirankumar Hullatti

**Affiliations:** Department of Pharmaconosy, KLE University’s College of Pharmacy, Belagavi, India

**Keywords:** Fractionation, column chromatography, preparative TLC, BSL bioassay, cell line studies

## Abstract

**Context:** Despite several phytochemical studies of *Premna serratifolia* Linn. (Verbenaceae), the isolation of active constituents of this plant remains to be explored.

**Objective:** The study isolates cytotoxic terpenoids and steroids from the leaves of *Premna serratifolia*.

**Materials and methods:** Unsaponifiable matter of hexane soluble fraction obtained from methanol extract was subjected to isolation by column chromatography and preparative TLC. Three compounds PS-01 A, PS-01B and PS-02 A were isolated. PS-01 A and PS-01B were identified by comparative TLC with authentic marker compounds followed by NMR analysis. Further PS-01B was analyzed by HR-GCMS. PS-02 A was subjected to HR-LCMS. All isolated compounds/fractions were evaluated for cytotoxic activity by BSL bioassay and using cell lines A549, HepG2 and L6.

**Results:** Three compounds were isolated from the leaf extract by bioactivity-guided fractionation. Two of which, namely, PS-01 A (oleanolic acid) and PS-02 A (unknown) were found to be terpenoids and PS-01B was identified as steroid (stigmasterol). PS-02 A compound is to be purified and characterized further. All three compounds PS-01 A, PS-01B, PS-02 A showed cytotoxicity by BSL bioassay (LC_50_ value of 54.49, 30.83, 16.32 ppm, respectively) and by cell line study where isolate PS-02 A has shown more cytotoxicity with LC_50_ values of 66.77 and 53.72 μg/mL with A549 and HepG2 cells, respectively, when compared with other isolates.

**Conclusion:** Bioactivity guided fractionation of *Premna serratifolia* leaves succeeded into isolation of two terpenoids and one steroid compound with significant cytotoxic activity. Here we report the isolation of these cytotoxic terpenoids/steroids from this plant for the first time which could be developed as anticancer agents.

## Introduction

*Premna serratifolia* Linn. (Verbenaceae) is a popular ingredient of Dashamoolas and is used in various Ayurvedic formulations due to its wide range of biological and therapeutic uses including antitumor, antidiabetic, hepato-protective, antiarthritic, anti-inflammatory, etc. (Yadav et al. 2010; Mali 2015). *Premna* is a scandent, erect shrub or small tree, more or less thorny on the trunk and large branches. Leaves are opposite or whorled and entire or serrate. The species is common along the Indian peninsular and Andaman coast. It is also distributed in the plains of Maharashtra, Gujarat, Assam, Khasi hills and Tarai (National Medicinal Plants Board 2008).

Terpenoids are the largest class of naturally occurring compounds having mainly cytotoxic properties. A large number of terpenoids exhibit cytotoxicity against a variety of tumour cells and cancer preventive as well as anticancer efficacy in preclinical animal models (Thoppil & Bishayee 2011). The anticancer activity of terpenoids appears promising and will potentially open more opportunities for cancer therapy (Huang et al. 2012).

As part of continuous studies on phytochemical investigations of *Premna serratifolia* leaves, the terpenoidal fraction was screened to identify and isolate the phyto-constituents seen on TLC profile and attempt was made to elucidate the structure of the isolates. Despite several phytochemical studies which proved the cytotoxic activity of leaf extracts, no reports were available on isolation based study. Methanol extract of *Premna serratifolia* leaves were found to have radical scavenging activity and tumour cell suppression potential (Selvam et al. 2012). Hence our focus was to isolate cytotoxic terpenoids from leaves extract to explore it for its anticancer potential.

## Materials and methods

### Plant material

Leaves of *Premna serratifolia* were collected from the premises of Regional Medical Research Centre (ICMR), Belagavi during January 2011 and authenticated by Dr. Harsha Hegde, Scientist ‘B’ ICMR, Belagavi, India. The voucher specimen of the plant (Accession Number RMRC-554) is deposited in ICMR Herbarium repository. Previously collected and authenticated plant material was used for the study.

### Extraction, fractionation and cytotoxicity by BSL assay

The powdered leaves were subjected to extraction with methanol. The extract was then fractionated according to the modified method adopted by Cos et al. (2006); Bhat et al. 2006; Hullatti et al. 2013. Cytotoxicity study was done for all sub fractions including isolated compounds by brine shrimp lethality (BSL) assay in accordance with the procedure adopted by McLaughlin and Rogers (1998). Phytochemical investigation was done for sub-fractions and isolates to confirm the presence of terpenoids/steroids by performing the chemical test of Liebermann Burchard Reaction according to the standard procedure (Sandjo & Kuete 2013; Bhat et al. 2006).

### Identification of the compounds

The number of compounds present in the sub-fractions obtained by column chromatography has been identified by thin layer chromatography (TLC). Optimized mobile phase i.e., toluene: ethyl acetate: glacial acetic acid (7:2:1) was used. Visualization of spot/band was done after derivatization with anisaldehyde-sulphuric acid spraying reagent.

### Isolation of the phytoconstituents

Based on the previous study results which proved the cytotoxicity of the fractions tested by BSL bioassay (Biradi & Hullatti 2014), only cytotoxic fractions, i.e., *n*-hexane soluble fraction of methanol extract was taken up for isolation of terpenoids/steroids. The *n*-hexane soluble fraction was subjected to saponification as per Ethiopian standard procedure (Ethiopian Standards Agency 2012). Unsaponifiable portion was taken up for silica based column chromatography. Open column chromatography was carried out using Silica gel 60. Preparative TLC was done using silica gel F_254_ and analytical TLC was done using pre-coated TLC plates coated with silica gel 60 F_254_. The different fractions were generated using gradient elution technique using petroleum ether, chloroform and acetone respectively by increasing the polarity gradually. The petroleum ether elution yielded PS-01 and chloroform/pet ether (1:4) elution yielded PS-02 sub-fractions. As per column fractionation done by Biradi and Hullatti (2015), a total 5 sub-fractions (SF) were generated from unsaponifiable portion (PS/F1/USM). The separation of all the sub fractions was established by TLC.

**Figure 1.**
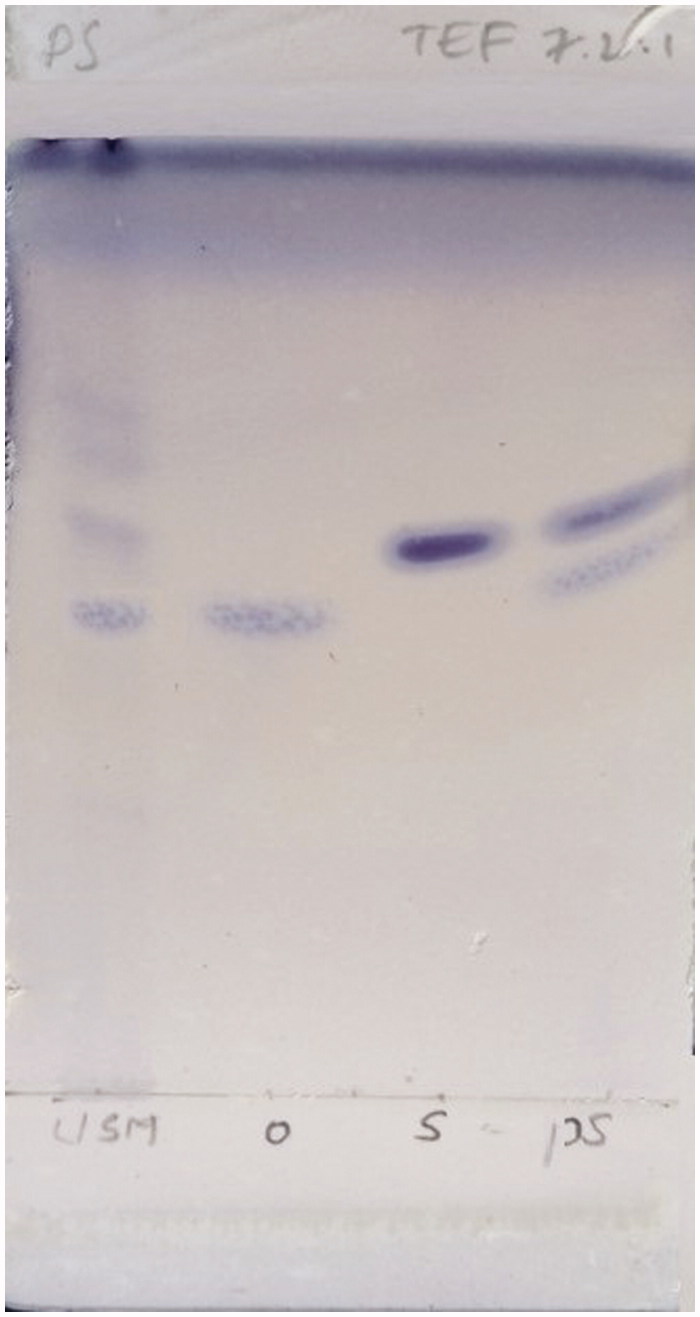
TLC of PS-01 MLR with markers.

**Figure 2.**
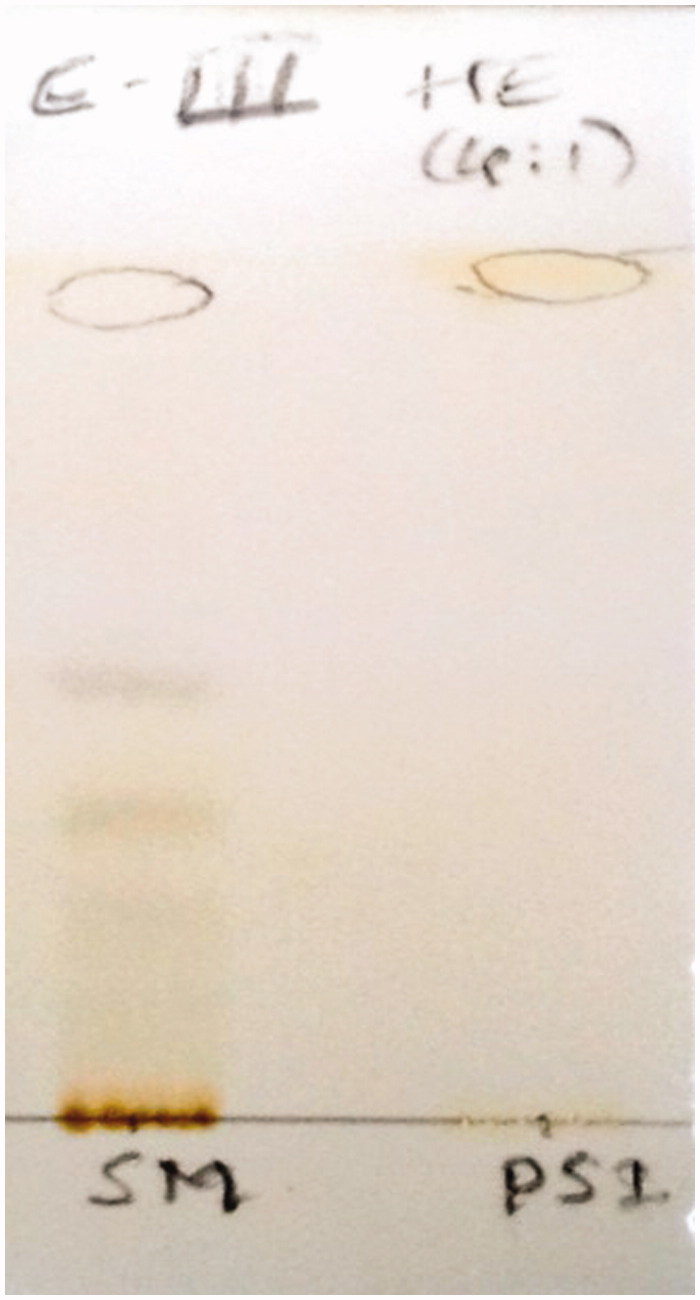
TLC of PS-02 A.

The methanol layer (MLR) obtained after processing/washing the isolated compounds PS-01 and PS-02 with methanol, were taken up for further purification on observation of TLC pattern where MLR of PS-01 was found to contain 2 bands with good resolution and MLR of PS-02 showed a single band near solvent front. The MLR of PS-01 fraction was subjected to comparative TLC with marker compounds oleanolic acid and stigmasterol available in the laboratory (Figure 1) where unsaponifiable matter (USM) was used as starting material of TLC and further subjected to preparative TLC for separation. The separation was confirmed again by TLC and coded as PS-01 A and PS-01B. Both the separated compounds exhibited the same colour and Rf when compared with the authentic standards. Then PS-01 A and PS-01B were subjected for characterization by HR-GCMS and NMR analysis to identify and confirm the structure. The MLR of PS-02 was coded as PS-02 A (Figure 2) was subjected to HR-LCMS.

#### Preparative TLC

A streak of crude fraction was applied manually on a preparative TLC glass plate (20 cm ×20 cm; 1 to 1.5 mm thickness). After air drying, the plate was developed in a pre-saturated glass chamber with mobile phase toluene: ethyl acetate: glacial acetic acid (7:2:1). Two plates were used in parallel where one was sprayed with ANS reagent and another was used to scrap off the separated bands carefully from the plate. The scratched sample was dissolved in chloroform and centrifuged at 3000 rpm for 5 min in order to remove silica. The supernatant was collected and filtered into separate Eppendorf tubes using 0.45 μm syringe filters. The tubes were kept open until the filtered supernatant dried completely. Then, the tubes were stored in desiccator at room temperature for further use (Rajauria & Abu-Ghannam 2013).

#### GC–MS analysis

Gas chromatography coupled with high resolution mass spectrometry (HR-GCMS) analysis was performed with an Agilent Technologies 7890 A gas chromatograph coupled to an AccuTOF GC-5 mass selective detector under the following instrumental conditions: HP-5 capillary column (30 m, 0.25 mm i.d., 0.25 μm film thickness) with oven temperature programed as initial temperature 40 °C for 1 min, then increased to 260 °C at the value of 15 °C/min for 5 min, final temperature 280 °C at the value of 20 °C/min. Head pressure was 18 psi; injector temperature: 270 °C; injection mode: 0.2 min splitless, volume injected 1 μL; detector temperature: 290 °C; carrier gas: helium. MS conditions: ion source temperature: 230 °C; electron impact: 70 eV; acquisition mode: scan (*m/z* 50–500).

#### LC-MS analysis

Liquid chromatography coupled with high resolution mass spectrometry (HR-LCMS) analysis was performed with an Agilent Technologies 6550 LC coupled to an iFunnel QTOF mass ESI detector under the following instrumental conditions: Column Details: Zorbax SB C18, 2.1 × 50 mm, 1.8 μ. Solvent A: 100% MilliQ Water +0.1% Formic Acid, Solvent B: 100% Acetonitrile +0.1% Formic Acid, Flow rate: 0.3 mL/min, Injection Volume: 3 μL, Run Time: 30 min, Elution mode: Gradient.

### Cytotoxicity of the isolated compound/fraction by Brine Shrimp Lethality (BSL) bioassay

Isolated compounds/fraction PS-01 A, PS-01B and PS-02 A were evaluated for cytotoxic activity by BSL bioassay according to method developed by McLaughlin and Rogers (1998). The brine shrimp (*Artemia salina* Lich.) eggs were procured from Department of Pharmacology, Manipal College of Pharmaceutical Sciences, Manipal, India. Brine shrimp eggs (50–60 mg) were sprinkled into darkened compartment of hatching chamber containing sea water and allowed to hatch at room temperature for 48 h. After hatching, the shrimps (nauplii) started to move toward smaller illuminated compartment through the holes made on compartment divider. About 10 nauplii were transferred into individual test tubes using pasture pipette along with sea water adjusting its final volume to 5 mL with sea water. A drop of dry yeast suspension was added to each test tube followed by test samples. Samples and standard (potassium dichromate) stocks were prepared in DMSO and further diluted with sea water to get final concentration of 10, 100, and 1000 μg/mL. Experiments were performed in triplicate for each concentration. Control tests were done by using equal volumes of distilled water. The test tubes were maintained under illumination. After 24 h, number of survivors were counted and the percentage of mortality and LC_50_ values were calculated by probit analysis using IBM SPSS Statistics for Windows, Version 20.0. (IBM Corp., Armonk, NY).

### Cytotoxicity of the isolated compound/fraction on A549, HepG2 and L-6 cell lines by MTT assay

A-549 (Human, small cell lung carcinoma), HepG-2 (Human, hepatic cancer) and L-6 (Rat, normal skeletal muscle) cell cultures were procured from National Centre for Cell Sciences, Pune, India. Test samples were dissolved in DMSO. Stock solution was prepared to final concentration of 1 mg/mL with Dulbecco’s Modified Eagle Medium (DMEM) in 2% fetal bovine serum (FBS) and filtered. The tests were performed in duplicate. The monolayer cell culture was trypsinized and the cell count was adjusted to 1.0 × 10^5^ cells/mL using DMEM containing 10% FBS. To each well of the 96 well microtitre plate, 0.1 mL of the diluted cell suspension was added. After 24 h, supernatant was flicked off upon formation of partial monolayer. The monolayer was then washed with medium and 100 μL of different test concentrations 62.5, 125, 250, 500, 1000 μg/mL of test drugs were added on to the monolayer in microtitre plates. The plates were then incubated at 37 °C for 3 days in 5% CO_2_ atmosphere and observations were noted every 24 h interval by microscopic examination. After 72 h, the drug solutions in the wells were discarded and 50 μL of MTT in PBS were added to each well. The plates were gently shaken and incubated again for 3 h at 37 °C in a 5% CO_2_ atmosphere. The supernatant was removed and 100 μL of propanol was added and mixed. The absorbance was measured using a microplate reader at a wavelength of 540 nm. The percentage growth inhibition was calculated using the following formula and concentration of test drug needed to inhibit cell growth by 50% (IC_50_) values was generated from the dose-response curves for each cell line (Danizot & Lang 1986)

$$\%\;Growth\;Inhibition=100-\left(\frac{Mean\;OD\;of\;individual\;test\;group}{Mean\;OD\;of\;control\;group}\times 100\right)$$

## Results and discussion

**Figure 3.**
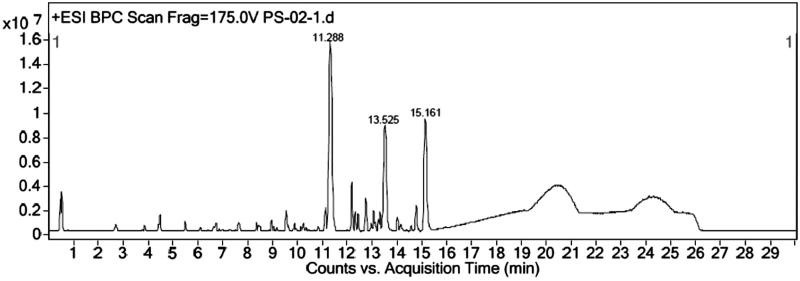
LCMS of PS-02 A.

**Figure 4.**
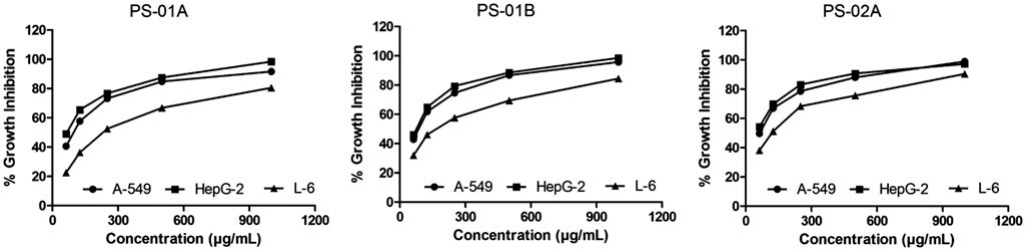
Cytotoxicity of the isolates on cell lines.

Unsaponifiable portion of *n*-hexane soluble fraction obtained from methanol extract was subjected to silica-based column chromatography followed by preparative TLC to yield three compounds. The PS-01 A and PS-01B are found to be known compounds which were identified by comparative TLC with known marker compounds oleanolic acid and stigmasterol. PS-01 A was obtained as light yellowish crystalline solid. It was found to be oleanolic acid (OA) upon comparing the TLC profile with authentic standard OA marker compound where both the test and standard compounds showed similar Rf value of 0.50 with same physical data and ^1 ^H and ^13 ^C NMR studies confirmed the structure. ^1 ^H-NMR (300 MHz, CDCl_3_): δ 0.79, 0.94, 1.00, 1.15, 1.27 (3 H, s), 1.64 (3 H, s, H-27), 2.86 (1 H, dd, H-18), 3.25 (1 H, dd, H-3), 5.30 (1 H, t, H-12). ^13 ^C-NMR (300 MHz, CDCl_3_): δ 77.01 (C-3). PS-01B was obtained as white crystalline solid. It was found to be stigmasterol by comparative TLC along with authentic stigmasterol marker and showed the same Rf (0.57). The compound was confirmed by matching the data of mass fragmentation coupled with gas chromatography (HR-GCMS) and National Institute Standard and Technology (NIST) data library and ^1 ^H and ^13 ^C NMR result supports the structure. HR-GCMS predicted the molecular formula as C_29_H_48_O. GCMS m/z (% relative abundance): 412(M+, 65), 397(14), 300(28), 271(16), 255(25), 213(9), 159(41), 145(37), 133(44), 105(46), 83(70), 55(100). ^1 ^H-NMR (300 MHz, CDCl_3_): δ 0.87 (d, 3 H), 1.05 (s, 3 H), 1.03 (s, 3 H), 3.62 (tdd, 1 H), 5.04 (m, 1 H), 5.13 (m, 1 H), 5.36 (t, 1 H). ^13 ^C-NMR (300 MHz, CDCl_3_): δ 140.76, 138.31, 129.28, 121.71, 71.81, 56.87, 55.96, 51.24, 50.17, 42.32, 42.22, 40.49, 39.69, 37.26, 36.52, 31.90, 31.68, 28.92, 25.40, 24.37, 21.21, 21.08, 19.40, 18.98, 12.24, 12.05. The sub fraction PS-02 A was obtained as pale yellow amorphous semisolid mass. The TLC showed the Rf of 0.97 and it was found to contain three major peaks/compounds by HR-LCMS spectral analysis (Figure 3). Among the three compounds, the compound at RT 11.288, 13.525 and 15.161 were found to have mass (*m/z*) of 573.25, 387.18 and 415.21 respectively. However, these compounds can be isolated in pure form by preparative HPLC with same chromatographic conditions used for LCMS method. All fractions/compounds were tested for cytotoxicity by BSL bioassay (Table 1) as well as on cell lines A-549, HepG2, and L-6 as normal which showed significant cytotoxicity (Table 2). The isolate PS-02 A has shown more cytotoxic effect with LC_50_ value of 66.77, 53.72 on A549, HepG2, respectively, as compared with normal cell line L-6 which shown LC_50_ of 112.93. Both cell lines showed significant cytotoxicity on isolated compounds as compared to normal cell line L-6. Graphical representation is shown in Figure 4.

**Table 1.** Cytotoxicity of the isolated compounds/fractions by BSL assay.

		Mean percent death after 24 h (concentration in μg/ml)			
Fraction code	Fraction name	10	100	1000	LC_50_ (PPM)
Control	Normal	0.00	0.00	0.00	0.00
Standard	Potassium dichromate	40	100	100	11.50
PS-01A	Oleanolic acid	23.33 ± 0.33	53.33 ± 0.33	96.67 ± 0.33	54.49
PS-01B	Stigmasterol	36.67 ± 0.33	66.67 ± 0.33	76.67 ± 0.33	30.83
PS-02A	Unknown	40	83.33 ± 0.33	100	16.32

Cytotoxicity effects (mean percent death after 24 h with LC_50_ values) of the plants are expressed in terms of mean ± S.E.M. of three replicates of samples.

**Table 2.** Cytotoxicity of the isolated compounds/fractions on cell lines.

Compound	IC_50_ (PPM)		
	A549	HepG2	L6
PS-01A	90.53	69.57	228.59
PS-01B	82.76	74.29	160.20
PS-02A	66.77	53.72	112.93

The compounds/fractions with IC_50_ values ≤100 μg/ml were considered as active.

This study reveals the isolation of cytotoxic terpenoids and steroids from *Premna serratifolia* leaves by bioactivity guided fractionation. Based on the previous study results, cytotoxic terpenoid and steroid fractions were selected for isolation of active compounds. Unsaponifiable matter obtained by saponification of fractions was considered for column chromatography study where we succeeded in isolation of three compounds (oleanolic acid, stigmasterol, and one unknown compound). Further isolated compounds were tested for cytotoxicity study on cell lines. The unknown compound PS-02 A showed more cytotoxic effect as compared with other known compounds. Although oleanolic acid and stigmasterol are abundantly present in most of the medicinal plants, the same have not yet been explored from *Premna serratifolia*. Hence, this is a new approach towards identification of phytoconstituents from this plant.

Recently, cytotoxicity screening on methanol extract of leaves, root bark and root wood of *Premna serratifolia* was performed using two cancer cell lines: B16 melanoma and SHSY-5Y neuroblastoma cells where a potent concentration-dependent cytotoxicity was shown by root bark extract whilst the leaves and root wood extracts did not show activity up to the highest concentrations tested (Habtemariam & Varghese 2015). Recent *in vitro* studies have revealed that the oleanolic acid and stigmasterol possesses the anticancer activity. Oleanolic acid induced a dose-dependent and time-dependent inhibition of HepG2 hepatocellular cancer cell growth mediated through arrest of the cell cycle, the induction of apoptosis and DNA fragmentation (Zhu et al. 2015). Stigmasterol has been reported to have chemopreventive effect on 7,12-dimethylbenz[*a*]anthracene (DMBA)-induced skin cancer investigated in Swiss albino mice (Ali et al. 2015).

In conclusion, bioactivity guided fractionation of *Premna serratifolia* leaves succeeded into isolation of two known compounds, i.e., oleanolic acid, stigmasterol, and one unknown impure compound. This study constitutes the first time isolation and identification of cytotoxic terpenoids and steroids from the leaves of *Premna serratifolia* plant which could be developed as anticancer agents.
